# Comparing energy-integrating detector and photon-counting detector-based breast cone beam CTs for microcalcification detection via Monte Carlo simulation

**DOI:** 10.1088/1361-6560/ae50ca

**Published:** 2026-03-23

**Authors:** Ahad Ollah Ezzati, Xiaoyu Hu, Miao Qi, Youfang Lai, Yuncheng Zhong, Kai Yang, Xun Jia

**Affiliations:** 1 Department of Radiation Oncology and Molecular Radiation Sciences, Johns Hopkins University, Baltimore, MD, United States of America; 2 Division of Diagnostic Imaging Physics, Department of Radiology, Massachusetts General Hospital, 55 Fruit Street, Boston, MA, United States of America

**Keywords:** Monte Carlo, breast CT, cone beam CT, photon counting

## Abstract

*Objective.* Microcalcification (*µ*Calc) detection plays an important role in breast cancer screening. Electronic noise in energy-integrating detectors (EIDs) is the major challenge for this task in current breast cone-beam CT (bCBCT) due to the tight dose constraint for breast imaging. bCBCT with a photon counting detector (PCD) can potentially offer a higher spatial resolution and lower noise. This study performed a direct comparison of bCBCTs with the two detector types via GPU-based Monte Carlo (MC) simulation. *Approach.* We employed Virtual Clinical Trial for Regulatory Evaluation toolkit to generate a realistic breast phantom with a 0.25 $\mathrm{mm}^3$ voxel size, 80% fat fraction and 14 cm diameter. We considered a bCBCT system with a 60 kV x-ray source filtered with 0.3 mm Cu and detector response functions for PCD and EID. A total of 360 projections were simulated with a total number of $3.15\times10^{12}$ photons, corresponding to ∼4 mGy mean glandular dose, comparable to a two-view mammography. We modified our GPU-based MC simulation code to incorporate analytical descriptions of *µ*Calcs of spherical shapes with diameters ranging from 0.1 to 0.4 mm, in 0.1 mm increments, into the voxelized phantom. A nichrome wire with 0.07 mm diameter was simulated to calculate the modulation transfer functions (MTFs). bCBCT images were reconstructed with the Feldkamp–Davis–Kress algorithm, and image quality and *µ*Calc detection performance were evaluated. *Main results.* EID-bCBCT had more profound image noise due to electronic noise. The image intensity standard deviations estimated within a region of interest were 0.055 cm^−1^ for EID-bCBCT and 0.038 cm^−1^ for PCD-bCBCT, respectively. *µ*Calcs and breast anatomy such as ligaments were more visible in the PCD-bCBCT images. The 10% MTF cutoffs were 5.5 and 9.5 lp mm^−1^ for EID-bCBCT and PCD-bCBCT, respectively. Contrast-to-noise ratio ranged in 1.20–9.13 for EID-bCBCT and 3.07–14.74 for PCD-bCBCT, depending on *µ*Calc sizes. *Significance.* We compared EID- and PCD-based bCBCT for *µ*Calc detection using GPU-based MC simulations in a clinically realistic setting. Our results demonstrate a potential advantage of PCD-bCBCT for this detection task.

## 1. Introduction

Breast cancer, accounting for 30% of all female cancers, is the most prevalent cancer among women (Bray *et al* 2024). Microcalcification (*µ*Calc) detection is a critical imaging task in breast cancer screening. Studies have shown that *µ*Calcs are associated with increased breast cancer risk, and particular patterns of them are correlated with probability of malignancy (Pang *et al* 2024). Hence, early detection and characterization of *µ*Calcs are important for timely intervention, allowing less invasive treatment options, and improving long-term outcomes for patients (Sardanelli *et al* 2017, Tot *et al* 2021).

Digital mammography (DM) is the standard method for breast cancer screening (Hong *et al* 2020). However, the projection nature of a 3D object onto a 2D plane impedes visualization of diagnostic information, especially with dense breasts. To overcome this challenge, 3D imaging techniques such as digital breast tomosynthesis (DBT) and breast cone-beam CT (bCBCT) (Ning *et al* 2007, Gazi *et al* 2015, Shah *et al* 2017) were developed. bCBCT offers fully tomographic imaging of breast anatomy, surpassing the quasi-tomographic imaging of DBT, at dose levels comparable to DBT and DM (Komolafe *et al* 2022). However, the limited power of *µ*Calc detection in bCBCT has been reported as its major challenge (Lindfors *et al* 2008, Aminololama-Shakeri *et al* 2016, Aminololama-Shakeri and Boone 2024). bCBCT imaging has conventionally used flat panel energy-integrating detectors (EIDs) (Ning *et al* 2007, Gazi *et al* 2015, Shah *et al* 2017). These detectors have additive electrical noise and are limited by scintillator blur and require relatively large pixel sizes (typically ${\unicode{x2A7E}}0.25\mathrm{-}0.5 \,\mathrm{mm}$) (Rajagopal *et al* 2021). For small-sized *µ*Calc detection, high spatial resolution and high signal-to-noise ratio (SNR) are equally critical. Due to the strong radiation dose constraints of breast imaging, to obtain a reasonable SNR under the inherent electrical noise, EID pixels must be binned (Yang *et al* 2010), leading to further loss in spatial resolution and impeding the performance in *µ*Calc detection.

Recently, as photon-counting technologies become mature and photon-counting detectors (PCDs) are implemented in clinical CT scanners, there is a growing hope that PCDs may have the potential to overcome the challenges associated with EIDs for the *µ*Calc detection task in bCBCT. Comparing to EIDs, PCDs have the advantages of relatively high spatial resolution and absence of electronic noise (Willemink *et al* 2018). Counting each photon based on its energy allows for energy discrimination, which can be used for spectral imaging and material decomposition, potentially benefiting visualization of *µ*Calcs and other subtle features in breast tissue (Yu *et al* 2016).

Before building a new bCBCT system with PCDs, it is a critical task to perform comprehensive simulation studies to understand the performance of PCD-bCBCT as compared to the conventional EID-bCBCT. Doing so is also important in exploring factors affecting the bCBCT system performance, such as imaging geometry, and deciding the optimal system design under realistic clinical scenarios. Over the years, Monte Carlo (MC) simulations have been extensively used to characterize the performance of CBCT systems (Verhaegen *et al* 1999, Park *et al* 2021, Mettivier *et al* 2022, Tomal *et al* 2025). To overcome the challenges in computation, GPU-based accelerations have been explored (Badal and Badano 2009, Jia *et al* 2012a, 2012b).

In this paper, we report our recent study comparing PCD- and EID-based bCBCT for the *µ*Calc detection task via MC simulation. To facilitate the computation, our previous GPU-based MC simulation tool for kV photon transport simulation in CBCT (Jia *et al* 2012a, Montanari *et al* 2014) was employed. We modified the transport code to enable the accurate modeling of a detailed breast phantom under voxelized geometry (Sharma *et al* 2019), while accommodating *µ*Calcs smaller than the voxel size using an analytical approach. We also incorporated realistic modeling of the imaging pipeline that may affect bCBCT performance, such as detector response, electronic noise in EIDs, and digitization of analog signals. Based on this computation pipeline, we investigated the performance of PCD-bCBCT for the *µ*Calc detection task and compared with that of EID-CBCT. Our preliminary study demonstrated the potential advantages of PCD-bCBCT in this targeted clinical application.

## 2. Methods

### 2.1. bCBCT setup

**Figure 1.**
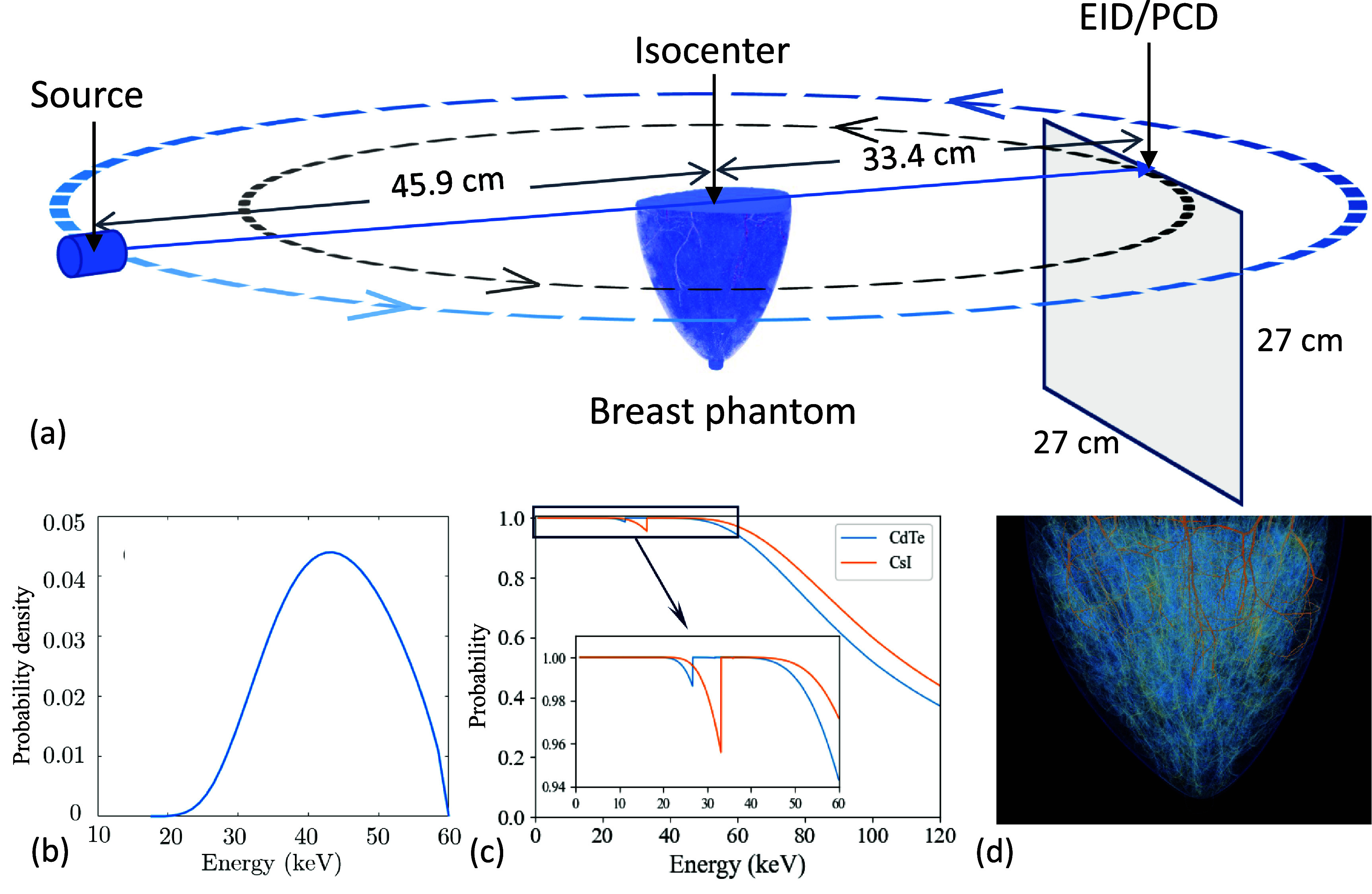
(a) Simulation setup including dimensions. (b) Energy spectrum of the x-ray source. (c) Response function of the PCD with 0.75 mm CdTe and EID with 1.0 mm CsI. (d) 3D rendering of the breast phantom. Note that the intensity of the image is set for rendering purpose only and does not represent physical meanings.

The geometry of the bCBCT scanner considered in this study is shown in figure 1(a). The vertical location of the x-ray focal spot was set at the top of the breast phantom at the chest wall. The source to isocenter distance was 45.9 cm and the source to detector distance was 79.3 cm (figure 1(a)). We considered a 60 kV tungsten x-ray source, filtered with 0.3 mm Cu, and with a focal spot size of $0.1 \times 0.1 \,\mathrm{mm}^2$. The x-ray spectrum was generated using the SpekCalc software (Poludniowski *et al* 2009) with a 20^∘^ anode angle (figure 1(b)). A flat x-ray fluence map covering the detector was considered for simplicity. The detector size was 27 cm in each dimension, and its pixel size was $0.1\times 0.1\,\mathrm{mm}^2$ for PCD and $0.2\times 0.2\,\mathrm{mm}^2$ for EID. In this study, detectors response functions (figure 1(c)) were considered, based on x-ray absorptions for the PCD with 0.75 mm CdTe and for the EID with 1.0 mm CsI. We considered 360 x-ray projections acquired within a full rotation.

We used the Virtual Clinical Trial for Regulatory Evaluation (VICTRE) tool (Sharma *et al* 2019) to generate an anthropomorphic breast phantom with $0.25^3~\mathrm{mm}^3$ resolution. A 3D rendering of the breast phantom is shownn in figure 1(d). The parameters of the configuration file were adjusted to create a pendent breast phantom with 80% fat fraction that represents the mean fat fraction of American women (Yaffe *et al* 2009). In order to generate continuous ligaments in the breast at the chosen voxel size, we adjusted the ligament thickness to 0.5 mm. The generated phantom had a semi-elliptical base (figure 1) with 14.6 and 13.6 cm axial and sagittal axes, respectively. The effective diameter of the breast was 14 cm, calculated by using Huang *et al* (2011) method, which corresponds to the most common diameter of the US female population (Boone *et al* 2004).

Each voxel had a density value and a material type defined by the VICTRE tool. Based on the material type, we assigned tissues’ x-ray attenuation properties for subsequent MC simulations. Tissue properties and density were assigned to each voxel according to the type. Table 1 shows different tissues of generated phantom and and their alternatives that were used in our simulations.

**Table 1.** VICTRE phantom tissues and their alternatives that were used in our MC simulations.

VICTRE phantom tissue	Material	Density ($\mathrm{g}\,\mathrm{cm}^{-3}$)
Air	Dry air	0.0012
Fat	Adipose tissue	0.92
Skin	Skin	1.10
Glandular, Nipple, Duct, Terminal Duct Lobular Unit	ICRP tissue	1.00
Muscle, Ligament	Striated muscle	1.04
Artery, Vein	Blood	1.06
Calcification	Calcium oxalate	2.12

### 2.2. MC simulation

#### 2.2.1. Overall MC simulation workflow

We employed the in-house developed gMCDRR (Jia *et al* 2012a, Montanari *et al* 2014) tool to simulate photon transport from the x-ray source to the detector. For each CBCT projection, gMCDRR first sampled the x-ray photons from the x-ray source based on the energy spectrum and fluence map. Then it rigorously transported photons from the source to the detector based on physics principles using the Woodcock algorithm (Woodcock *et al* 1965). This method considers photon transport in a homogeneous medium with the total x-ray attenuation coefficient being the maximum total attenuation coefficient, $\mu_{\mathrm{max}}(E)$, for the entire simulation volume. It samples the distance to the next interaction site using the total attenuation coefficient. This approach effectively allows photon transport simulation without the need for complex ray tracing or checking voxel boundary crossings. To incorporate the variability in actual x-ray attenuation coefficients across voxels, a fictitious attenuation coefficient of *µ*_max_(E) - $\mu_{\mathrm{i}}(E)$ is assigned to each voxel. At each interaction site, an interaction type is sampled among the real events, i.e. Compton scattering, Rayleigh scattering, photoelectric absorption, or the fictitious event, based on their respective x-ray attenuation coefficients. In a fictitious event, the photon’s energy and direction remain unchanged, while for physical interactions, they are sampled following the physics model for the corresponding interaction type. This process continues until the photon goes outside the phantom or with energy lower than the threshold of 1 keV. No variance reduction technique was used in our simulation.

For photons that exited the phantom and subsequently hit the detector, rejection sampling using the detector response function as the rejection function (figure 1(c)) was conducted to determine whether the signals should be recorded. If so, the pixel location was first identified based on the coordinate of the photon on the detector, and the detector signal was updated. For EID, energy of the photon was then recorded to the corresponding pixel. For PCD, the photon count was increased by one. The photon cut-off energies for EID and PCD were set to 5 and 20 keV, respectively. Photons below these cut-off energies were discarded. For each photon hitting the detector, we repeated the rejection sampling and signal recording twice, each for the PCD and the EID case, respectively. Additionally, the gMCDRR code was modified to score primary and scattered photons separately, so that we could investigate the impact of the scatter photons on the task of *µ*Calc identification. In addition, we also tallied the total energy that was deposited in each voxel of the phantom from all the projections. Physical dose of each voxel was then computed by dividing the energy with mass of the voxel. The mean glandular dose (MGD), mean skin dose (MSD), and mean breast dose (MBD) for the whole breast (skin included) were calculated by averaging dose values within the corresponding anatomical structures.

The total number of photons in the MC simulation is an important parameter governing the simulation realism and validity of the results, as it is directly related to dose and thus influences image quality and noise characteristics. The *µ*Calc detection task is particularly challenging under low-dose conditions due to the excessive noise, making it crucial to perform the simulation at an appropriate dose level to faithfully represent clinical scenarios (Boone *et al* 2005). reported the air-Kerma required to deliver MGD equal to two-view mammography (4 mGy), for different breast sizes and fat fractions. They also reported photon fluence per mGy of Air-Kerma of the x-ray tube at the isocenter. Based on the data, we used linear interpolation to calculate the required Air-Kerma for a breast with 14 cm diameter and 80% fat fraction. We further obtained the number of photons in our simulation by multiplying this Air-Kerma with photon fluence per mGy of Air-Kerma and the field size at the isocenter, yielding $3.15\times10^{12}$ for all 360 projections.

MC simulations were performed on a desktop workstation equipped with an Intel Xeon Silver 4214R CPU and an NVIDIA RTX A4000 GPU. Including *µ*Calc spheres in the transport simulations substantially increased runtime; accordingly, we recorded the computational (wall-clock) time as a function of the number of *µ*Calcs.

#### 2.2.2. *µ*Calc and wire object insertion

We inserted eight *µ*Calc clusters in the breast phantom, with each cluster including four *µ*Calcs. Each of the *µ*Calc was modeled as a sphere, defined by its coordinate of the center of the sphere and the corresponding radius. The diameter of the four *µ*Calcs in a cluster were 0.4, 0.3, 0.2 and 0.1 mm. To facilitate visual identification of them in the phantom images, these *µ*Calcs were arranged at the four corners of a square with a side length of 2.5 mm. We placed two clusters in each of the four slices located at 1.75, 4.75, 7.75 and 10.75 cm away from the chest wall. At these distances, the cross section size of the breast vary, representing different levels of difficulties in identifying the *µ*Calcs due to different x-ray attenuations and amount of scattering signals. Within each slice, one cluster was placed in the central region and the other in the periphery region of the breast.

The voxelized breast phantom had $0.25^3~ \mathrm{mm}^3$ voxel size and the inserted *µ*Calcs diameters ranged in 0.1–0.4 mm. Thus, these *µ*Calcs cannot be accurately represented by the voxelized geometry. It is not preferable to reduce the voxel size of the phantom due to memory limitation. To overcome this challenge, the gMCDRR photon transport kernel was modified to allow the incorporation of *µ*Calcs described by an accurate geometry to model *µ*Calcs with sizes similar to or smaller than the voxel size. Specifically, in the Woodcock transport scheme, a key operation is identifying the material properties at the photon’s location. For voxelized geometry, this is accomplished by determining the voxel containing the photon and retrieving the corresponding material properties. In the presence of *µ*Calcs, additional checks were implemented to determine whether the photon position lies within any spherical *µ*Calc regions, based on distances to sphere centers and their corresponding radii. If so, the calcium oxalate cross section and density were used. The remaining transport simulation steps were unchanged. The approach effectively overlayed the *µ*Calc spheres on top of the voxelized representation of the breast phantom.

Note that Woodcock transport scheme requires that the transport simulation is conducted in a homogeneous phantom with the total x-ray cross section value being at least the largest one that exists in the phantom. Under the modification of including *µ*Calcs in the phantom, the total cross section value became that of a *µ*Calc. This increase in total x-ray cross section reduced the mean free path of photons in simulations. This fact, along with the repeated check of photon positions relative to each of the *µ*Calc spheres, reduced the simulation efficiency, which will be characterized in this study.

To assess image resolution, we also simulated a nichrome wire (80% nickel and 20% chrome) with a density of 8.2 g$\,\mathrm{cm}^{-3}$ and 0.07 mm diameter in air to enable the calculation of modulation transfer function (MTF). The wire was located in the isocenter, tilted by 3^∘^ with respect to the rotation axis of the scanner. The same approach as modeling the *µ*Calc spheres was used to model the wire to enable the precise modeling of the wire as a cylindrical object in MC simulations.

### 2.3. Electronic noise and signal digitization for EID

To model the electronic noise for EID, we estimated the noise level characterized by the noise signal standard deviation (SD) in eV based on the information for a CsI-based flat panel detector system (PaxScan 4030CB, Varian Medical Systems, Palo Alto, CA). Specifically, according to Kyprianou *et al* (2009), the exposure at which the electronic noise equals the quantum noise at 90 kV was $R = 0.2~\mu$Roentgen. To utilize this information to estimate electronic noise, we first estimated the photon fluence corresponding to this exposure level, and then estimated the quantum noise level corresponding to this photon fluence. As such, we generated a 90 kV spectrum using SpekCalc software (Poludniowski *et al* 2009) at the 20^∘^ anode angle, denoted as $\psi(E)$, and estimated the photon flux that hits the detector at this exposure level as

\begin{align*} \phi = \frac{R\times0.00877\left(\frac{\mathrm{Gy}}{\mathrm{Roentgen}}\right)}{\int \mu_{\mathrm{tr}}\left(E\right)\psi\left(E\right) E \, \mathrm{d}E} = 2111650~\mathrm{cm^{-2}}, \end{align*}

where $\mu_\mathrm{tr}(E)$ is the energy-transfer coefficient. Considering the EID response function *H*(*E*) in figure 1(c), the flux of the recorded photons was calculated as

\begin{align*} \phi^{^{\prime}} = \phi\int \psi\left(E\right) H\left(E\right) \, \mathrm{d}E = 2061200~\mathrm{cm^{-2}}. \end{align*}

For the EID pixel size of 0.2 mm, the number of photons in each pixel and hence, the related quantum noise SD was 824 and 28.7, respectively. We further computed the mean photon energy recorded by the detector corresponding to the 90 kV spectrum, yielding 39 keV. Thus, the SD of the electronic noise was estimated as $28.7\times 39 = 1120$ keV.

To model this electronic noise, Gaussian noise with this SD and zero mean was added to each pixel value of the EID after MC simulation. Negative values of the resulting pixel values were truncated to zero.

To model the digitization process converting analog signal to digital signal for EID, we used the reference point of 9000 digital value for an air area in the projection image with a 0.2 mm pixel pitch to derive the analog to digital units (ADUs) (Yang *et al* 2010). In our simulation, the average recorded energy in air area per pixel was ${\sim}2.4\times10^{8}$ eV. Hence, the calculated energy step for digitization was $2.4\times10^{8}/9000 = 22730$ eV/ADU. For a simulated EID projection, the recorded energy per pixel was digitized with this scalar, rounding to the nearest integer.

### 2.4. Image reconstruction

We reconstructed volumetric images for both the PCD-bCBCT and EID-bCBCT cases using a high spatial resolution with a voxel size of $0.1^3 \,\mathrm{mm}^3$. This fine voxel sampling was chosen to fully leverage the intrinsic spatial resolution of the simulated systems and to enable detailed visualization of anatomical structures as well as noise characteristics. For the PCD case, we additionally applied a $2 \times 2$ pixel binning to the simulated projection images prior to reconstruction. This step effectively increased the detector pixel size from $0.1 \times 0.1 \,\mathrm{mm}^2$ to $0.2 \times 0.2 \,\mathrm{mm}^2$, which better matches the voxel size in the reconstructed volume, considering the approximately twofold geometric magnification of the imaging setup. This binning strategy also helps reduce image noise and data size while preserving sufficient spatial resolution to match the reconstruction grid.

The volumetric reconstructions were performed using the standard Feldkamp–Davis–Kress algorithm (Feldkamp *et al* 1984) implemented on a GPU platform to ensure computational efficiency. A Shepp–Logan kernel was employed during the filtered backprojection step to balance spatial resolution and noise suppression. This GPU-based implementation enabled the reconstruction of high-resolution 3D volumes within a practical time frame, facilitating direct comparison between the PCD- and EID-based bCBCT images.

### 2.5. Evaluations

In addition to visually assessing the image quality of the resulting bCBCT images, we compared EID and PCD-bCBCT images using quantitative metrics. A region of interest (ROI) was selected, see figure 3, to calculate the SD of the PCD and EID-bCBCT images, as a surrogate for overall image noise. At known *µ*Calc positions, we extracted ROI with a size of $75\times75$ pixels to visually compare the visibility of the *µ*Calcs. We then computed contrast-to-noise ratio (CNR) of each *µ*Calc. Specifically, voxels overlapping the *µ*Calc region were identified as the foreground, while the remaining voxels within the ROI were designated as the background. Contrast was defined as the difference in mean pixel intensities between the foreground and background regions, and noise was estimated as the SD of pixel intensities within the background region. We note that, because the *µ*Calc size is smaller than or comparable to the voxel size, partial volume effects introduce uncertainty into the resulting CNR calculation. To evaluate spatial resolution, we calculated the MTF of the bCBCT images using the nichrome wire. As such, an ROI around the isocenter with a size of $5\times19$ pixels was selected. This ROI allowed us to obtain the oversampled line spread function (LSF), and the MTF was computed by the Fourier transform of the LSF (Fujita *et al* 1992).

## 3. Results

### 3.1. Absorbed dose distribution

**Figure 2.**
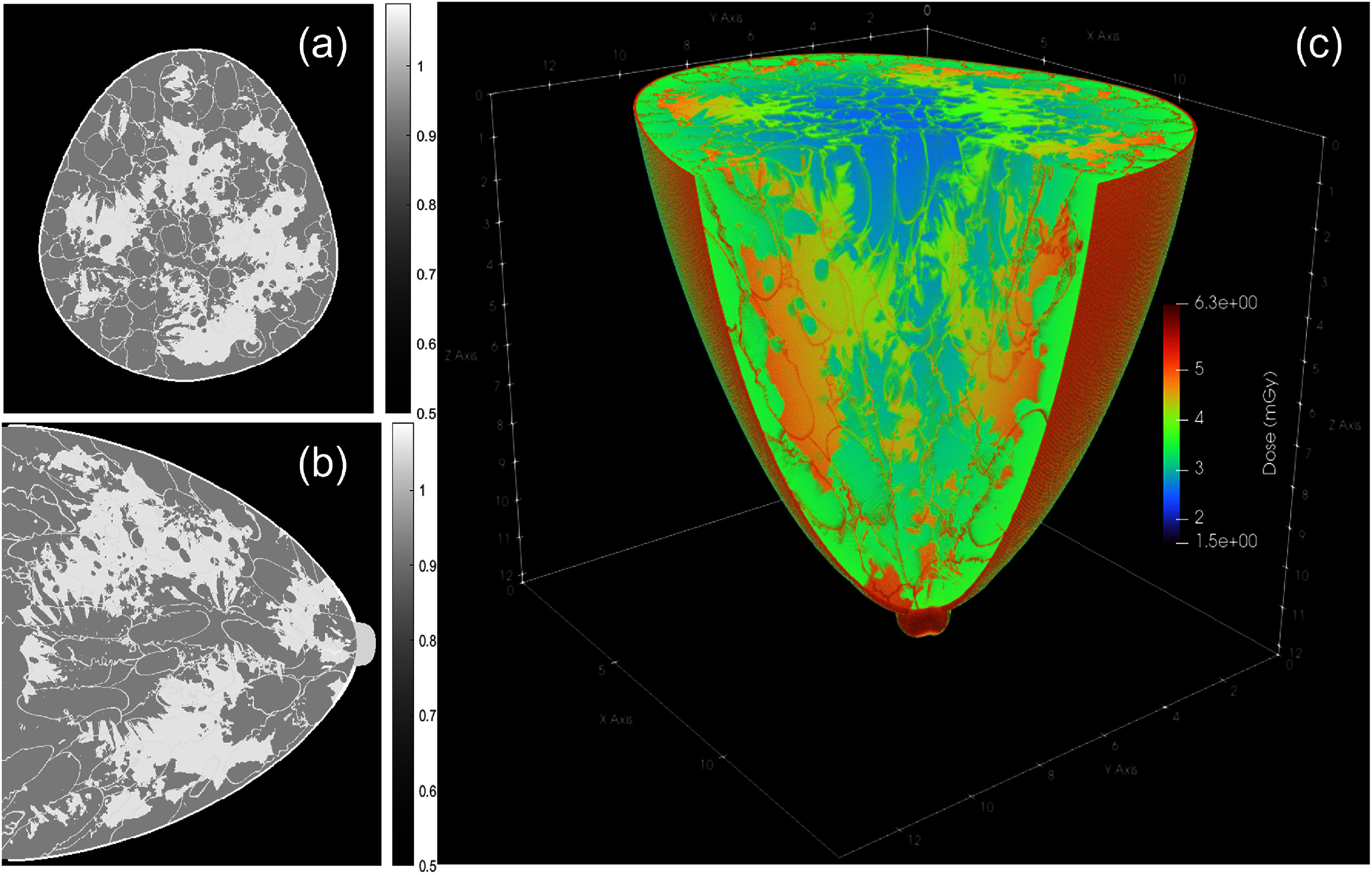
(a) Coronal and (b) sagittal density images (unit g cm^−3^). (c) 3D dose distribution.

Figures 2(a) and (b) present the density in representative coronal and sagittal slices. In figure 2(c), we present the 3D view of the dose distribution in the breast. The dose was generally higher in the periphery region. The calculated dose values for MBD, MGD without skin, MGD with skin, and MSD were 3.6, 4.3, 4.4, and 5.5 mGy, respectively.

### 3.2. Comparison of PCD and EID-bCBCT images

**Figure 3.**
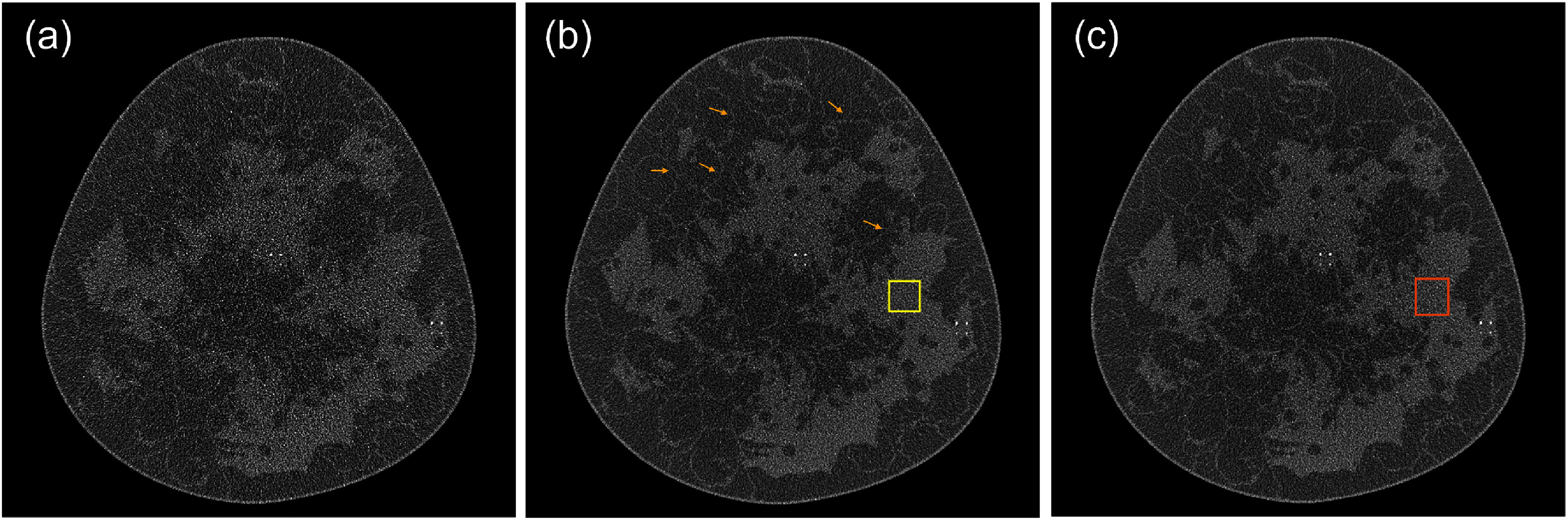
Representative reconstructed EID- and PCD-bCBCT images (display window: [0.1–0.4] cm^−1^). (a) EID-bCBCT image reconstructed using all photons. (b) PCD-bCBCT image reconstructed using all photons. Square is the ROI for calculating SD. Arrows indicate fine structures preserved in PCD-bCBCT image but lost in EID-bCBCT image. (c) PCD-bCBCT image reconstructed using primary photons only.

Figures 3(a) and (b) show the reconstructed images for the PCD and EID cases in a representative slice located 7.75 cm from the chest wall. More anatomical details, such as ligaments, can be clearly visualized in the PCD-bCBCT images compared to the EID-bCBCT images, as pointed out by the arrows. The higher noise level in the EID-bCBCT images is the primary factor contributing to the loss of fine structural details (e.g. ligaments) in these reconstructions. The square overlay indicates the ROI used for noise SD calculation. The measured SD values for the PCD- and EID-bCBCT images were 0.038 and 0.055 cm^−1^, respectively.

**Figure 4.**
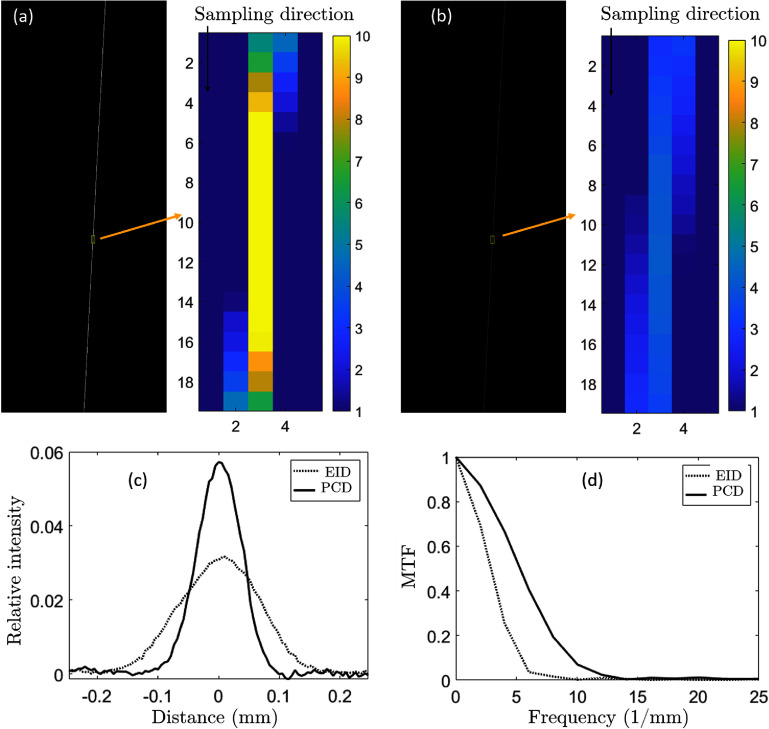
Nichrome wire in the reconstructed (a) PCD-bCBCT and (b) EID-bCBCT images. Display window is to [1, 10] cm^−1^. (c) Fine sampled LSFs. (d) Calculated MTFs.

Figure 4 shows the nichrome wire in the reconstructed volumetric images for the PCD and the EID cases. For better visibility, the ROIs used for MTF calculation are shown in color including the sampling directions in figures 4(a) and (b). Given the 3-degree angle between wire and scanner rotation axis and the images voxel size 0.1 $\mathrm{mm}$, the distance between the fine sampling points was calculated as $\tan(3^\circ) \times 0.1 = $ 0.005 24 mm. Figure 4(c) shows the fine sampled LSFs for the PCD and the EID cases using the Fujita *et al* (1992) method. The fast Fourier transforms of the LSFs were calculated and plotted, yielding MTFs in figure 4(d). The 10% MTF cutoffs were 5.5 and 9.5 lp mm^−1^ for EID and PCD-bCBCT, respectively.

### 3.3. Detectability of *µ*Calcs

**Figure 5.**
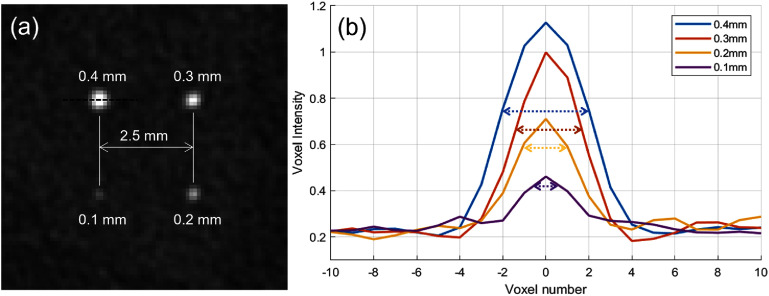
(a) Image of a reconstructed PCD-bCBCT slice around a *µ*Calc cluster. Display window is [0.1, 0.4] cm^−1^. (b) Voxel intensity profile along the center of each *µ*Calc, as illustrated by the dash line in (a). Arrows indicate the actual size of the *µ*Calcs.

Figure 5(a) shows a PCD-bCBCT image of a *µ*Calc cluster located near the center of the field, 10.75 cm from the chest wall. Voxel-intensity profiles drawn through the centers of individual *µ*Calcs are plotted in figure 5(b). The peak widths in these profiles (e.g. full width at half maximum, FWHM) generally scale with the true diameters of the *µ*Calcs. However, for the smallest *µ*Calcs (e.g. 0.1 mm diameter), the measured FWHM exceeds the actual size, indicating limited spatial resolution of the imaging system.

**Figure 6.**
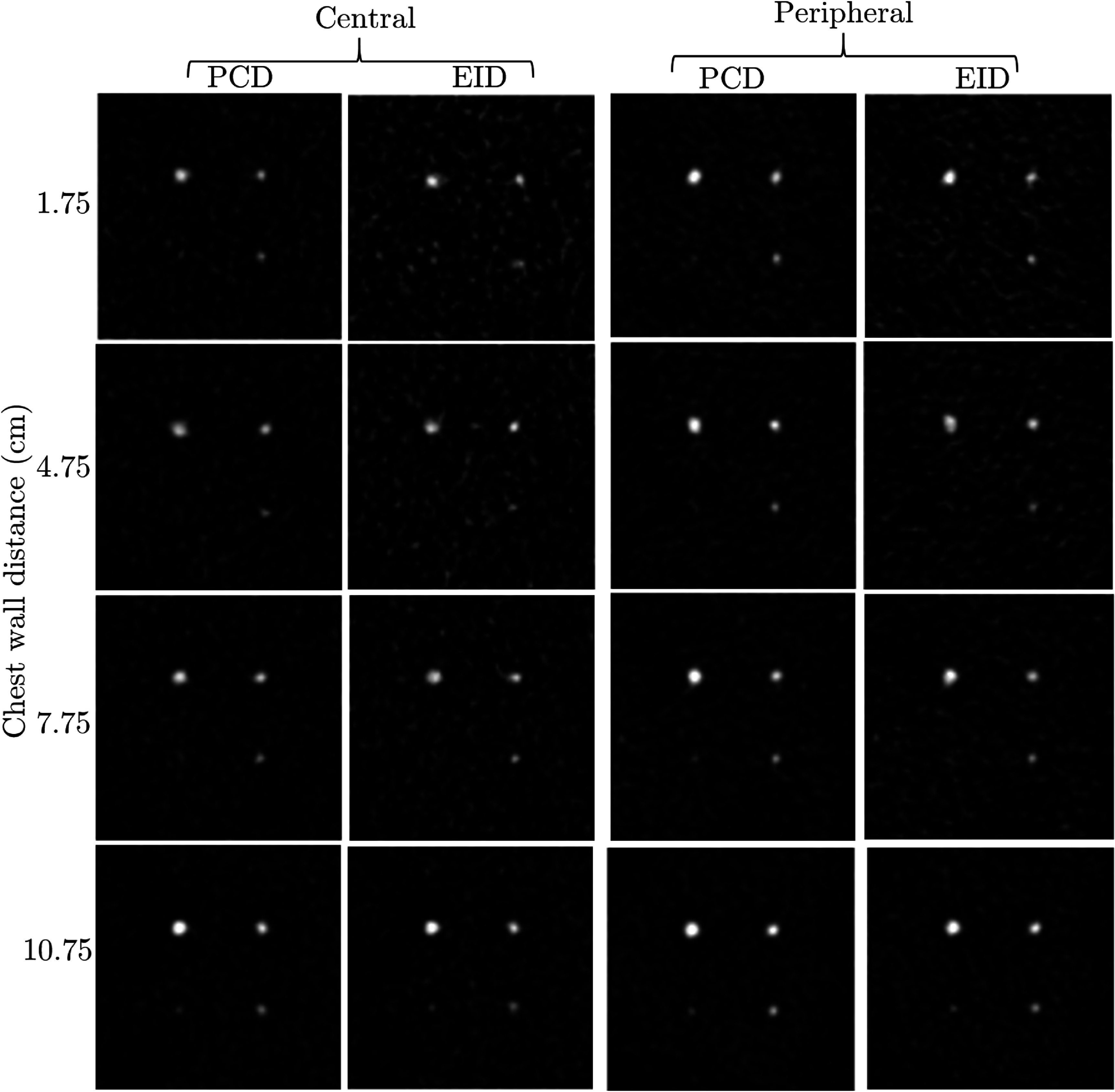
Reconstructed bCBCT images of eight *µ*Calcs clusters at four different distances from the chest wall, and at the central region and the peripheral region of the breast. Display window [0.2, 1.0] cm^−1^.

Figure 6 presents zoomed in images for all the eight *µ*Calc clusters at four different distances from the chest wall, and at the central region and the peripheral region of the breast. Generally, EID-bCBCT images are noisier than PCD-bCBCT images. *µ*Calcs with diameters greater than or equal to 0.2 mm were visible in all images. For *µ*Calcs with 0.1 mm in diameter, they are more visible in PCD-bCBCT images than in EID-bCBCT images.

Quantitatively, in the first three rows of table 2, we present CNR of EID-bCBCT and PCD-bCBCT *µ*Calcs, and the ratio between them. Each number is the average of over all the *µ*Calcs at different locations of the corresponding size. Generally, CNR for PCD-bCBCT was found higher than that of the EID-bCBCT. Averaging over *µ*Calcs of all sizes, CNR with PCD-bCBCT was 80% higher than that with EID-bCBCT. The ratio was found to be particularly higher for the *µ*Calcs with 0.1 mm in diameter.

**Table 2.** CNR of *µ*Calcs that were detected by primary and all photons.

*µ*Calc size (mm)	0.4	0.3	0.2	0.1
EID-bCBCT	9.13	7.77	6.70	1.20
PCD-bCBCT	14.74	12.54	9.54	3.07
CNR$_\mathrm{PCD}$/CNR$_\mathrm{EID}$	1.61	1.61	1.42	2.55
PCD-bCBCT (Primary photons)	16.57	14.06	10.64	3.54

### 3.4. Impact of scattered photons

**Figure 7.**
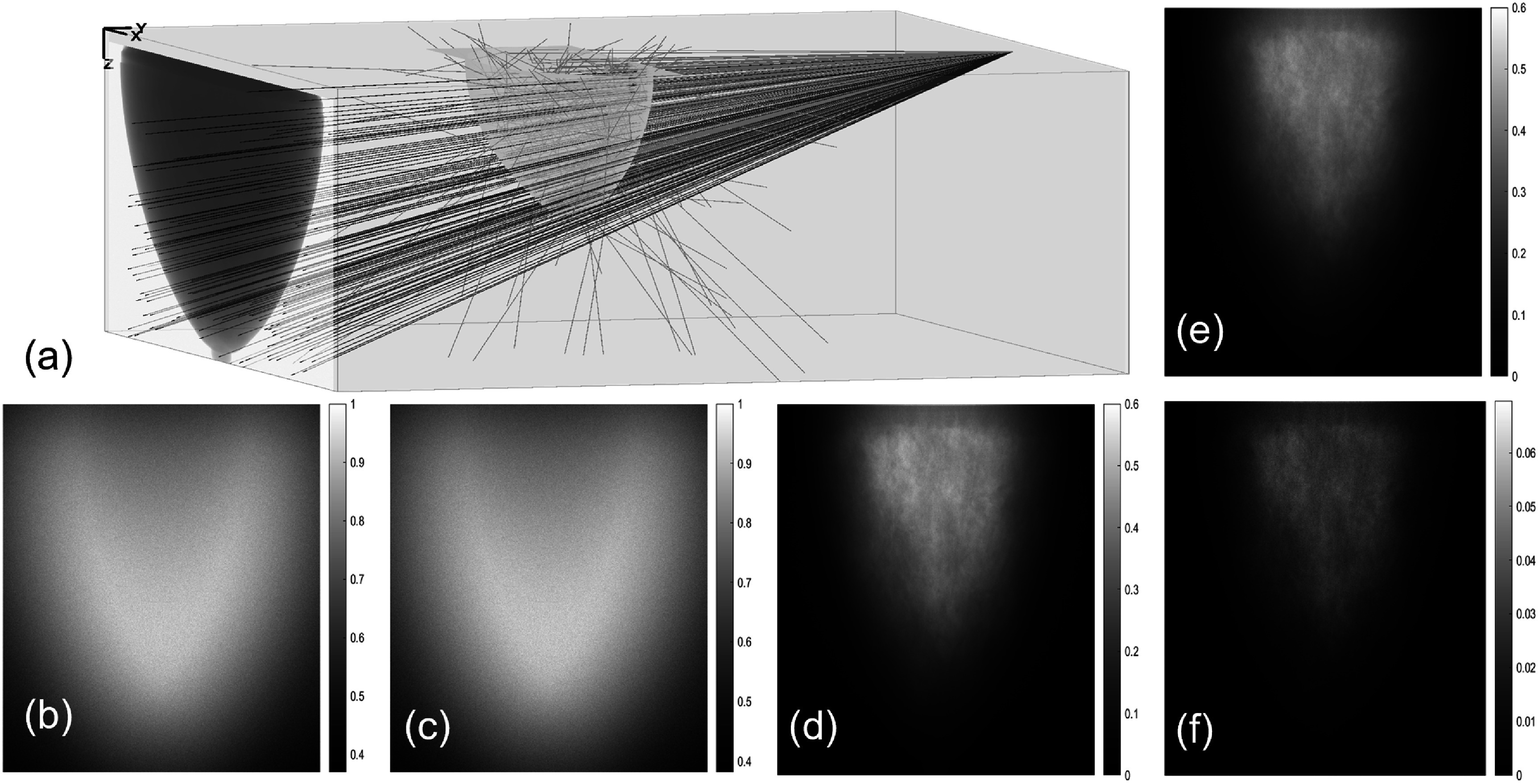
(a) Illustration of photon tracks in one example bCBCT projection. (b) and (c) Scattered photons intensity in PCD and EID normalized to corresponding maximum value. (d) $\mathrm{SPR}_{\mathrm{PCD}}$. (e) $\mathrm{SPR}_{\mathrm{EID}}$. (f) $\mathrm{SPR}_{\mathrm{PCD}}$–$\mathrm{SPR}_{\mathrm{EID}}$.

Figure 7(a) illustrates photon transport through the breast phantom. In figures 7(b) and (c), we present the scattered photon signal recorded in one example projection in the PCD-bCBCT and the EID-bCBCT cases, respectively. Both were normalized to the range of [0, 1.0]. The scattered photon signal appeared to be weaker in the center of the breast region than that around the breast boundary due to photon attenuation at the center region. Figures 7(d) and (e) depict the scattered-to-primary ratio (SPR) of the two cases, and figure 7(f) presents the difference between them. Different from the scatter signal distribution, SPR generally was higher in the center region than the boundary region due to the lower primary photon signal in the center region. The maximum value of SPR for both images were 0.6, with the SPR of PCD-bCBCT slightly higher.

To demonstrate the impact of scattered photons, we considered a hypothetical situation that the PCD-bCBCT image is reconstructed using only primary photons. Figure 3(c) shows the reconstructed slices at 7.75 cm chest wall distance using all photons. Comparing with the images reconstructed with all the photons (figure 3(a)), scattered photons introduced cupping artifact that reduces image contrast. It was also observed that the image reconstructed with all photons had a reduced noise level due to reduction in quantum noise. SD of the ROI for the image with all photons and that of the primary photons were 0.038 and 0.043 cm^−1^, respectively.

We also included in table 2 the average CNR values for the *µ*Calcs in PCD-bCBCT images reconstructed using only primary photons. The CNR was slightly higher compared to that of the images reconstructed with all photons. Between these two settings, the primary-photon-only reconstruction exhibited both increased contrast and higher noise; however, the improvement in contrast outweighed the noise increase, resulting in an overall enhancement in CNR.

### 3.5. Simulation time

The total time for the entire simulation and image reconstruction process was approximately 62 h, with the majority of this time consumed by the MC simulation.

Compared to the simulation without *µ*Calcs inserted, the simulation time increased due to two facts. The first one was the use of calcium cross sections in the Woodcock tracking algorithm to allow modeling the *µ*Calcs. The efficiency of the Woodcock algorithm in MC simulations is influenced by the disparity between the maximum cross section used in the simulation and the actual physical cross sections. A higher maximum cross section results in a shorter mean free path in simulation, thereby increasing the number of virtual interactions required to transport a photon through the geometry. Based on our study, performing MC simulation using the *µ*Calc cross sections in the Woodcock tracking algorithm but without *µ*Calcs physically inserted increased the simulation time by a factor of 2.7.

**Figure 8.**
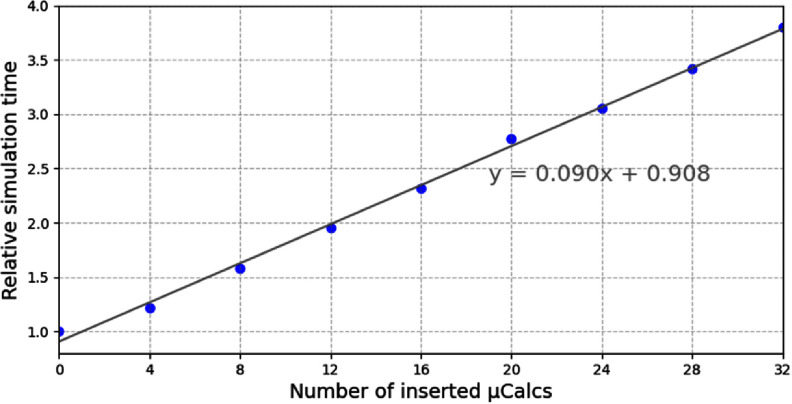
Relative MC simulation time as function of number of *µ*Calcs.

Second, explicitly modeling the *µ*Calcs as spherical objects within the simulation geometry leads to an additional increase in computation time, due to the boundary checks performed at each Woodcock step. Figure 8 shows the relative simulation time as a function of the number of inserted *µ*Calcs with respect to the simulation time without *µ*Calcs but using the calcium cross sections. As expected, the simulation time scales approximately linearly with the number of *µ*Calcs.

## 4. Discussions

### 4.1. GPU-based MC simulation platform

In this work, we developed a comprehensive MC simulation pipeline to model the entire bCBCT imaging process, facilitating an efficient preliminary investigation on the potential advantages of PCD-bCBCT for *µ*Calc detection in a clinically realistic setting while achieving high computational efficiency. Here ‘clinical’ refers to the use of realistic imaging geometry, clinically relevant dose levels, practical numbers of projections, anthropomorphic breast structure, realistic *µ*Calc sizes, and explicit modeling of x-ray scatter and detector response. This end-to-end framework enables a faithful representation of the imaging chain from x-ray generation to reconstructed images. Our MC simulation platform includes both PCD and EID models in a single unified simulation framework, avoiding the need to run separate simulations for different detector types. The photon transport module of the gMCDRR code was modified to support the insertion of *µ*Calcs smaller than the phantom voxel size and to tally primary and scattered photons separately, enabling accurate modeling of signal and noise characteristics. The intention was to reflect the key physical and operational constraints of breast CBCT acquisitions rather than to reproduce a specific vendor reconstruction or correction pipeline. Although simplified elements were present, as is typical in physics-based simulation studies, the framework is expected to capture the dominant factors governing detectability and enable meaningful comparison between detector technologies under clinically relevant conditions.

A major technical advance of this study is the development of a hybrid voxelized-analytical photon transport scheme within a GPU-based MC framework, enabling accurate simulation of sub-voxel *µ*Calc while retaining the computational efficiency offered by GPU acceleration to simulate a realistic number of photons, on the order of 10^12^, corresponding to a MGD of approximately 4 mGy. The calculated dose results supported the appropriateness of the chosen photon number in our MC simulation, which is a critical parameter for ensuring realistic dose conditions. Such large-scale simulations can also be performed using CPU-based or cluster computing resources. However, GPU acceleration offers a practical approach to enable these simulations to be carried out with greater accessibility. This capability enables us to not only capture realistic noise properties and signal statistics, both of which are essential for assessing *µ*Calc detectability under clinically relevant dose conditions, but also to compute the dose distribution within the breast with fine spatial detail in the computational phantom, e.g. in figure 2. This level of dosimetric precision allowed for validating simulation realism and for supporting subsequent studies on dose optimization and imaging performance evaluation.

We did not explicitly estimate the statistical uncertainty of the MC simulations, as the photon number was chosen to represent a realistic clinical imaging scenario. Under this setting, the resulting noise characteristics, such as the image intensity SD measured in reconstructed images, reflect statistical fluctuations in the simulations but are expected to be representative of those encountered in clinical bCBCT. Consequently, the reported noise levels characterize clinically relevant imaging performance rather than MC sampling uncertainty.

Beyond the scope of this initial study, the developed simulation platform provides a powerful tool to support a wide range of subsequent investigations. These include optimizing bCBCT system hardware design, evaluating the impact of contrast agents, and performing large-scale virtual clinical trials to quantitatively assess new imaging modalities. By enabling realistic and efficient MC simulations of bCBCT, this work establishes a valuable foundation for advancing breast imaging research.

### 4.2. Advantages of PCD-bCBCT

The detection and characterization of *µ*Calcs in breast imaging is a challenging task, particularly in low-dose bCBCT where image noise strongly degrades detectability. In such dose-constrained scenarios, the signal associated with small *µ*Calcs is often buried in background noise, making reliable identification difficult even with advanced reconstruction techniques. Our study demonstrated advantages of PCD-bCBCT over conventional EID-bCBCT for *µ*Calc detection under realistic low-dose conditions.

An important factor contributing to the observed CNR differences between EID- and PCD-based CBCT systems is the fundamentally different signal formation mechanisms of the two detector technologies. In EIDs, the detected signal is proportional to the total deposited energy, which results in higher weighting of high-energy photons, particularly after beam hardening through the breast. This energy weighting reduces effective material contrast between *µ*Calcs and surrounding tissue and can also contribute to cupping artifacts in reconstructed images, both of which degrade CNR. In contrast, PCDs count individual photons and can preserve energy-dependent attenuation differences, leading to improved contrast retention for high-*Z* structures such as *µ*Calcs. Meanwhile, the superior performance of PCD-bCBCT can also be attributed to the absence of electronic noise. In EID systems, electronic noise exists in detector readout, which can dominate the overall noise profile, especially at low signal levels typical of low-dose imaging. In contrast, PCDs register individual photon events without additional electronic noise contributions, thereby improving the CNR and enhancing the visibility of small, high-frequency structures such as *µ*Calcs. This noise advantage has been studied extensively (Yu *et al* 2016, Willemink *et al* 2018), and our study further confirms it in the *µ*Calc detection task. Furthermore, the fine intrinsic spatial resolution of PCDs and their energy discrimination capability offer additional benefits for imaging small, high-contrast features. Although these aspects were not the primary focus of the present study, they are expected to further improve *µ*Calc detection performance and will be investigated in future work.

The CsI scintillator thickness used for the EID model influences detector detection efficiency and, consequently, image quality. In this study, we chose a 1.0 mm CsI layer thickness, rather than the more commonly used value 0.6 mm (Colbeth *et al* 2005, Maschio 2015), to ensure that the EID and PCD exhibit comparable detection efficiencies, as shown in figure 1(c). This study was not meant to represent a specific commercial system, but to ensure a fair and conservative comparison across detector technologies. Additionally, for the 60 kV beam, the photon absorption fraction was 0.995 for 1.0 mm CsI and 0.970 for 0.6 mm CsI. This minor difference was not expected to significantly affect the comparative EID performance observed in this study. Even under this conservative configuration that favors EID performance by increasing detection efficiency, PCD-bCBCT demonstrated advantages over EID-bCBCT in *µ*Calc detection. We anticipate that these advantages would be further amplified for EID systems employing thinner CsI layers.

Overall, our findings highlight the potential of PCD-bCBCT to overcome some of the fundamental noise limitations associated with EID-based systems in low-dose breast imaging. These results support the continued development and evaluation of PCD technology as a promising pathway for improving *µ*Calc detection and characterization in bCBCT.

### 4.3. Impact of scattered photons

The peak value of the SPR was found to be 0.6, with that of the PCD-bCBCT being slightly higher. Shah *et al* (2017) calculated the SPR for bCBCT scanner with 50 and 30 cm source-to-isocenter and isocenter-to-detector distances, respectively. They found SPR of 0.8 for a phantom with 15 cm diameter, which is in agreement with our calculated value.

The effect of scattered photons on *µ*Calc detection was also investigated in this study by separately recording projection data with and without scatter contributions. No scatter correction was applied in this analysis. It was found that the reconstructed image using only primary photons exhibits higher noise compared to the image reconstructed with all photons. This increase in noise arises from the reduced number of detected photons, which leads to higher quantum noise. However, excluding scattered photons also enhances image contrast, as scatter typically contributes a low-frequency background. Overall, the contrast enhancement outweighs the increase in noise, resulting in an improved CNR. As shown in table 2, removing scattered photons leads to an approximately 13% increase in the CNR of *µ*Calcs. The fact that reduction of scatter in breast CT improves *µ*Calc visibility agreed with previous studies (Ghazi 2020). This study provided a physics-based first step to understand factors affecting *µ*Calc detectability and to inform future system development, rather than to replicate a specific clinical bCBCT pipeline nor to present the final theoretical upper limit of *µ*Calc detectability of PCD-bCBCT.

### 4.4. Limitations of the study

This study has several limitations. First, the x-ray source was modeled as a uniform point source, and the anode heel effect was not included. While this simplification is unlikely to affect the relative EID–PCD comparison at identical locations, it may influence position-dependent image quality. In clinical bCBCT systems, the heel effect introduces spatial variations in fluence and spectrum along the chest wall–nipple direction, which can affect noise, contrast, and CNR. Consequently, spatial variations in image quality may be underestimated in this study.

This study modeled the detector response function but did not account for other detector-related effects, such as pixel cross talk, charge sharing, pulse pileup, or dead time, which can influence image quality and detection performance, particularly for PCD systems. While the simplified detector model enables a controlled comparison of EID- and PCD-based bCBCT under idealized conditions, these unmodeled effects may degrade image resolution, increase noise, or alter contrast in practical implementations. Consequently, the absolute image quality and *µ*Calc detectability reported here may differ from those observed in clinical systems. Incorporating more comprehensive detector physics in future simulations will allow a more complete assessment of detector-specific performance.

## 5. Conclusion

In this study, we performed a direct comparison of PCD- and EID-based bCBCT under low-dose imaging conditions using MC simulations. To enable this comparison, we employed a GPU-based MC simulation pipeline that realistically models the bCBCT imaging process. The pipeline extended our previous gMCDRR tool to support accurate modeling of *µ*Calc geometry within a voxelized anthropomorphic breast phantom and incorporated detector-specific energy response functions. This simulation framework provides a flexible tool for investigating advanced breast x-ray imaging technologies. Our results indicate an advantage of PCD-bCBCT for *µ*Calc detection, attributable primarily to the absence of electronic noise and improved contrast, which together enhance visualization of fine structures at clinically relevant dose levels.

## Data Availability

The data that support the findings of this study are available upon reasonable request from the authors.
